# Xenobiotic Exposure and Autoimmune Hepatitis

**DOI:** 10.1155/2010/248157

**Published:** 2010-12-30

**Authors:** Kathleen M. Gilbert

**Affiliations:** Arkansas Children's Hospital Research Institute, University of Arkansas for Medical Sciences, 13 Children's Way, Little Rock, AR 72202, USA

## Abstract

Although genetics contributes to the development of autoimmune diseases, it is clear that “environmental” factors are also required. These factors are thought to encompass exposure to certain drugs and environmental pollutants. This paper examines the mechanisms that normally maintain immune unresponsiveness in the liver and discusses how exposure to certain xenobiotics such as trichloroethylene may disrupt those mechanisms and promote autoimmune hepatitis.

## 1. Immunological Characteristics of Autoimmune Hepatitis

Autoimmune hepatitis (AIH) is a disease characterized by progressive liver inflammation of unknown etiology that may advance to fibrosis. The inflammation encompasses both cell-mediated cytotoxicity by infiltrating lymphocytes and the production of autoantibodies. Although not restricted to AIH, many patients with AIH make autoantibodies specific for asialoglycoprotein receptor (ASGPR) [1] and alcohol dehydrogenase (ADH) [2]. Type 1 AIH is characterized by circulating antinuclear antibodies (ANA) and smooth-muscle antibodies (SMA) [3]. Some individuals may have antineutrophil cytoplasmic autoantibodies (ANCA), antibodies to soluble liver antigens or liver pancreas (anti-SLA/LP). Type 2 AIH is associated with antibodies against liver-kidney microsome 1 (LKM-1) and/or antibodies against liver cytosol 1 antigen (LC1) [4]. LKM-1 autoantibodies react with linear epitopes within cytochrome P450 2D6 (CYP2D6), a phase-I drug- and toxicant-metabolizing enzyme in the liver, and perhaps major antigen target of type 2 AIH. 

Diagnosis of AIH usually involves more than the measurement of autoantibodies since patients may express them intermittently or produce antibodies that are not part of the standard repertoire. As described in Table 1 a definitive diagnosis of AIH is multifactorial. One classic indicator of AIH is liver pathology associated with lymphocyte infiltration of portal region. The liver infiltrate includes macrophages, antibody-secreting plasma cells, and T lymphocytes of both CD4^+^ and CD8^+^ subsets. Several investigators have reported a predominance of CD4^+^ T cells in the liver infiltrate, while others have reported a predominance of CD8^+^ T cells [5–7]. Regardless of the exact cell makeup the periportal lymphocyte infiltration characteristic of AIH differs from other autoimmune liver diseases such as primary biliary cirrhosis and autoimmune cholangitis in which lymphocytes instead target the bile ducts. 

The specificity of the T cells that infiltrate the liver in AIH is still being defined. Using T cell clonal analysis, it was found that the majority of clones generated from the peripheral blood of patients with type 1 AIH were TCR*αβ* CD4^+^ T cells, while most of the clones obtained from the liver were TCR*γδ* CD4^−^CD8^−^ T cells or TCR*αβ* CD8^+^ T cells [8]. Both types of liver-derived T cell clones proliferated in response to ADH and ASGPR. In patients with type 2 AIH, both CD4^+^ T cells and CD8^+^ T cells that proliferated and produced IFN-*γ* in response to stimulation with CYP2D6 have been generated from liver tissue and peripheral blood [9, 10]. Further study of T cell receptor variable *β*-chain transcripts suggested that the T cells that mediate pathology in type 2 AIH are oligoclonal [11] and that different effector types target different epitopes of CYP2D6 [12]. 

Regardless of their specificity, it is not clear how activated liver-infiltrating T cells escape deletion or tolerance to become effector cells in AIH. The liver normally demonstrates very little inflammation and is extraordinarily easy to transplant. Several mechanisms have been proposed to explain the anti-inflammatory nature of liver tissue. The hyporesponsiveness may be due in part to the intrahepatic entrapment and deletion or tolerance of activated T cells, including liver-specific T cells, by liver sinusoidal endothelial cells (LSEC) or Kupffer cells [13, 14]. In addition, it has been proposed that constitutive engagement of Toll-like receptors by gut-derived microbial molecules leads to low level but constant production of IL-10 [15], which in turn suppresses the activity of T cells and NK cells that would otherwise mediate the inflammation characteristic of AIH [16, 17]. Signaling by another anti-inflammatory cytokine TGF-*β* has similarly been found to mediate liver hyporesponsiveness [18]. Lastly, T_Reg_ cells also appear to help maintain immune tolerance in the liver [19]. The development of AIH in humans presumably requires a defect in one or more of these normally efficient methods of preventing T cell-mediating tissue destruction in the liver. For example, patients with active AIH have been shown to be defective in the number and activity level of T_Reg_ cells [20, 21] and in their expression of TGF-*β* receptor type II on peripheral blood mononuclear cells [22]. Taken together, the normal immune hyporesponsiveness in the liver can be attributed to a network of related mechanisms, one or more of which must be disrupted for the development of inflammation associated with AIH. 

Treatment of AIH usually involves long-term administration of anti-inflammatory or immunosuppressive drugs such as prednisone and/or azathioprine. Depending on the definition of a response, up to 35% of AIH patients are refractory to treatment [23]. Among those patients that respond to therapy, the ten-year survival rates decrease from 94% to 62% if cirrhosis is present at diagnosis [24]. Since early stages of AIH are often asymptomatic, 25% of patients have already progressed to cirrhosis by the time the disease is first diagnosed [25]. The gap of several years that can exist between disease initiation and diagnosis makes it especially difficult to identify the events that trigger pathogenesis. 

## 2. Genetic Contribution to AIH

Several susceptibility factors for AIH disease development have been identified, even if the mechanistic bases for their contributions are still unclear. Similar to most autoimmune diseases, AIH predominates in women at a ratio of 3.6 : 1 [26]. Racial, regional, and genetic predisposition can also affect the clinical manifestations of AIH [23]. Studies to delineate genetic susceptibility in AIH have largely focused on genes within the human leukocyte antigen (HLA) region. In Europe and North America increased susceptibility to type 1 AIH is associated with the HLA-DR3 and HLA-DR4 serotype [3]. However, the HLA haplotypes considered as risk factors for type 1 AIH change in other regions of the world, leading to the supposition that different genetic associations are present in different populations and that the peptide specificity of the T cells in these populations also differs (for review see [27]). Susceptibility to type 2 AIH is often associated with the HLA-DR7 and DR3 haplotypes. 

Increased susceptibility to AIH can also be conferred by polymorphisms in non-HLA genes. Although these genes include some not directly involved in immune function (e.g., thiopurine S-methyltransferase) [28], most involve genes associated with immune responsiveness. For example, a polymorphism in the gene for the adhesion molecule cytotoxic T lymphocyte-associated antigen 4 (CTLA-4) has been shown to increase susceptibility to a number of autoimmune conditions, including type 1 AIH [29, 30]. Also, described as possible risk, factors for AIH include polymorphisms in the genes for TNF-*α* [31] and complement component C4 [32]. Polymorphisms in genes that control apoptosis (e.g., death receptor Fas; CD95) have similarly been shown to promote AIH [33]. Since activation-induced apoptosis is an important mechanism by which the host protects itself from autoreactive T cells, AIH susceptibility factors associated with apoptosis may be particularly important mechanistically. 

Twin studies examining concordance rates have not been reported in patients with AIH. Consequently, it is not clear exactly how much genetics contributes to disease development. Concordance rates reported in other autoimmune diseases vary considerably but are rarely more than 25% [34–36]. Thus, although genetics contributes to autoimmunity, disease development is primarily attributable to one or more environmental factors.

## 3. Environmental Contribution to AIH: Drugs

Autoantibodies specific for phase I and phase II metabolizing enzymes can be found in patients with either type 1 or type 2 AIH. This has led to the suggestion that some compounds that perturb these enzymes (e.g., certain drugs and xenobiotics) should be considered as potential environmental triggers of AIH. Most ingested pharmaceuticals and xenobiotics are metabolized in the liver into more active, and in some cases toxic, breakdown products. The phase I metabolizing enzymes, for example, cytochrome P450s (CYPs) account for approximately 2–4% of total liver protein. In humans the most abundant CYP isotype is CYP3A (29%) followed by CYP2C (18%), CYP1A2 (13%), and CYP2E1 (7%). CYP2D6 is only found in small amounts (<2%), but accounts for the metabolism of almost 30% of all drugs [37]. 

A recent retrospective review of patients diagnosed with autoimmune hepatitis in the Mayo clinic showed that in 9.2% of the cases disease development could be attributed to drug treatments [38]. Drugs most often associated with the development of AIH include nitrofurantoin, halothane, and minocycline [38, 39]. More recently, a review of case reports has associated treatment with statins or infliximab with the development of AIH [40, 41]. Although several other types of drug-induced liver injury appear to be immune mediated, it is not clear that these injuries can be classified as AIH. 

Autoantibodies specific for different P450s have been identified in the sera from individuals with drug-induced hepatitis and leading to the hypothesis that these enzymes play a role in disease etiology. For example, autoantibodies against CYP2E1 were found in patients suffering from halothane-induced hepatitis [42], and anti-CYP1A2 antibodies and anti-CYP2C9 were found in patients with dihydralazine-induced or tienilic acid-induced hepatitis, respectively [43]. It is not clear how or why CYPs stimulate an immune response. Although drugs are substrates for CYPs, the former can also regulate the levels and activity of the later. Even at subtoxic doses, several drugs including acetaminophen, the anesthetic urethane, and members of the selective serotonin reuptake inhibitors class of antidepressants have been shown to regulate different types of CYPs, in some cases increasing and in other cases decreasing activity [44–47]. 

The regulatory effects of drugs on CYPS may be due in part to adduct formation that alters the activity of the enzymes. Several drugs are converted by CYPs into reactive metabolites that form adducts with liver proteins that have been identified as, or at least comigrated with, CYPs. For example, trifluoracetylated CYP2E1 was found in the liver of rats exposed to halothane [48]. Similarly, the antihypertensive drug dihydralazine has been shown to form adducts with its primary metabolizing enzyme CYP2A1 [49]. It seems logical that the protein most likely to interact with a drug reactive metabolite would be the enzyme involved in the formation of that metabolite. It has been suggested that the chemically induced adduct formation increases the immunogenicity of the metabolizing enzymes and thereby promotes autoantibody production to the altered protein. Most drug-metabolizing P450s are localization in the ER membrane of hepatocytes, a site not particularly accessible to immune cells. However, some P450s can also be found, albeit at much lower levels, in other cellular locations such as mitochondria and plasma membrane. CYP2D6, CYP1A1, and CYP2E1 have been detected at the cell surface by flow cytometry or biochemical analysis of the plasma membrane [50].The molecular mechanisms responsible for targeting and transport of P450s to these alternative intracellular sites appear to be dependent on the cellular systems and experimental conditions used. Thus, exposure to certain drugs and xenobiotics may promote the surface expression of CYPs, as well as enhance their immunogenicity through adduct formation. The resulting antibodies could interact with P450s as antigenic targets in the plasma membrane and cause immune-mediated destruction of the hepatocyte. 

Polymorphisms in P450s may help account for interindividual variation in susceptibility to toxicity induced by drugs and other xenobiotics. In terms of drugs CYP2D6, 2C19, and 2C9 polymorphisms account for the largest variation in response since most drugs are metabolized by these enzymes; however, polymorphisms in other P450 genes such as CYP1A1 and 2E1 have been noted. With regard to other xenobiotics and liver disease, the role of P450 polymorphisms in disease susceptibility is far from clear. Polymorphisms in CYP2E1 have been correlated with increased likelihood of liver lesions in Chinese workers exposed to vinyl chloride [51]. Specific CYP1A2 polymorphisms have been shown to modify smoking-related hepatocellular carcinoma [52]. On the other hand, other studies found no link between well-studied single-nucleotide polymorphisms (SNPs) in CYP2E1 and alcoholic liver disease or chronic benzene poisoning [53, 54]. Genome-wide association studies (GWAS) instead of a focus of individual P450 genes may be required to more clearly identify gene-environment interactions.

## 4. Environmental Contribution to AIH: Pollutants

The mechanisms by which drugs may promote an immune response to metabolizing enzymes in the liver could also apply to certain toxicants. However, since xenobiotic exposure often occurs without our knowledge, such exposure is often much more difficult to document than contact with drugs. Even in those cases when exposure to pollutants is documented, it almost always involves multiple chemicals, making it difficult to describe a cause/effect relationship with a particular pollutant. For these reasons, evidence of toxicant-induced AIH is mostly based on the results of animal studies.

Carbon tetrachloride (CCl_4_) is an organic compound that was once used extensively as a cleaning fluid, refrigerant, and insecticide. However, once its toxicity became apparent its use was largely phased out in the USA. The liver is the organ system most sensitive to CCl_4_ toxicity, due in part to the inherently high rate of CYP2E1-mediated metabolism of the toxicant in this tissue. CCl_4_ is a very stable chemical, and it has accumulated in water and soil thanks to its once wide-spread release by domestic manufacturing and processing facilities [55]. Today, most nonoccupational human exposure occurs via air and water. In animal systems CCl_4_ has been mostly used to generate acute toxicant-induced hepatitis. However, CCl_4_ at lower doses can induce fibrosis following chronic administration [56]. The liver pathology of CCl_4_ has been associated with at least transient increases in IFN-*γ*, TNF-*α*, and TGF-*β* [57]. Alterations in adhesion molecules (e.g., ICAM-1 and PECAM-1) were also observed in the livers of CCl_4_-treated rats. In addition, co-exposure to carbon tetrachloride was shown to augment the generation of a nonpathogenic autoimmune response against liver protein in mice infected with mouse hepatitis virus A59 [58]. Thus, it seems that CCl_4_ at least augments the development of chronic T cell-mediated AIH.

## 5. Trichloroethylene and AIH

Another environmental pollutant with a more defined connection to AIH is trichloroethylene (TCE). TCE is an organic solvent that was widely used as a metal degreaser. Reports of possible TCE toxicity have led to its replacement by supposedly less harmful compounds. However, because of improper disposal techniques, TCE has become a major environmental pollutant found in air, water supplies, and soil. TCE is the most frequently reported organic contaminant in groundwater [59], which is the source of 93% of public water systems in the USA. 

Although the exposure to TCE is widespread, it rarely occurs at levels thought to be directly toxic. However, a number of reports have linked low-level chronic TCE exposure, either occupational or environmental, to a variety of autoimmune diseases in humans including systemic lupus erythematosus, scleroderma, bullous pemphigoid, and diabetes [60–68]. There are also links between TCE exposure and autoimmune liver diseases. For example, statistically significant clusters of individuals listed for liver transplantation with a diagnosis of autoimmune hepatitis or primary biliary cirrhosis were identified in association with US EPA monitoring sites recording mean daily levels of chlorinated hydrocarbons in the 90th percentile [69]. TCE was found to be a major contaminant at such sites. 

Skin problems, mostly irritant contact dermatitis due to the defatting action of the solvent, are a common problem for occupational users of TCE. However, a different type of idiosyncratic hypersensitivity skin disorders associated with TCE use has become the main clinical issue in the past 20 years in Asia [70]. Over 90% of the patients suffering from this TCE-induced generalized hypersensitivity also suffer from nonviral, immunologically induced hepatitis, and in many cases increased levels of total IgG [71]. Use of a guinea pig model confirmed that TCE can cause two pathophysiologically different types of the hepatitis. The first is the well-known toxic liver injury caused by high level-TCE exposure, while the second is immune mediated and is induced by TCE doses below those causing toxic liver injury [70]. 

Even if overt autoimmune disease is not diagnosed, signs of immune activation including increased numbers of T cells have been associated with chronic exposure to a domestic water supply contaminated with TCE [60, 72–74]. Similarly, occupational exposure to TCE resulted in significantly increased serum levels of the T cell-derived pro-inflammatory cytokine IFN-*γ* [75]. Taken together, these results provide strong circumstantial evidence that exposure to TCE can promote T cell hyperactivity and autoimmunity in humans, and this autoimmunity can manifest itself in different types of autoimmune disease, including those that target the liver.

## 6. Mouse Model of TCE-Induced AIH

Based on the epidemiological data we used a mouse model [76] to more directly study the link between TCE and autoimmune disease. This mouse model utilizes autoimmune-prone MRL+/+ mice since a genetic propensity for autoimmunity is thought to be required for idiopathic as well as experimental autoimmune disease. We found that female MRL+/+ mice exposed to low, occupationally relevant concentrations of TCE in their drinking water (2.5 mg/mL) for 32 weeks, developed AIH characterized by plasma cell and T cell infiltration of the periportal region of the liver [77, 78]. A more recent study showed that exposure to an even lower concentration of TCE (0.5 mg/mL) for an even shorter time period (26 weeks) similarly initiated pathology commensurate with autoimmune hepatitis [79]. TCE-induced AIH was accompanied by a time-dependent increase in the number of antibodies specific for liver microsomal proteins. TCE exposure in mice also expanded the percentage of activated IFN-*γ*-secreting CD4^+^ T cells. Taken together these results showed that TCE exposure induced AIH, complete with lymphocyte infiltration and generation of antiliver antibodies, in MRL+/+ mice.

## 7. TCE Metabolism and AIH

**Figure 1:**
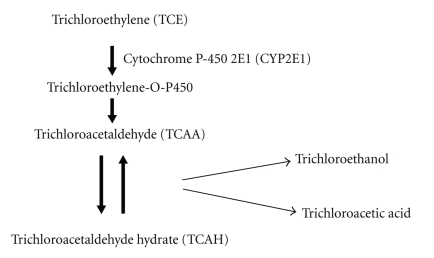
Oxidative metabolism of TCE.

Determining whether a xenobiotic requires metabolism to mediate its toxicity can provide important mechanistic clues. Once ingested, some TCE is stored in adipose tissue, but most TCE is quickly distributed to the liver. There it undergoes oxidative metabolism that is primarily mediated by CYP2E1. CYP2E1 rapidly converts TCE to an intermediate (trichloroethylene-0-P450) that can rearrange to trichloroacetaldehyde (TCAA) which in solution is in equilibrium with trichloroacetaldehyde hydrate (TCAH) (Figure 1). TCAA and TCAH are then converted to trichloroacetic acid or trichloroethanol which is excreted as the alcohol glucuronide [80]. The TCE metabolism pathway in humans is similar, albeit slower, to that of mice [81].

Acute toxicity of high-dose TCE requires its metabolism [82]. A similar requirement for the autoimmune-promoting effects of low-dose TCE was investigated. Blocking CYP2E1 activity inhibited the ability of low-dose TCE to alter T cell activity [83], suggesting that it was a downstream metabolite of TCE that actually promoted the T cell-mediated autoimmunity of AIH. To test this possibility TCAH, instead of TCE, was added to the drinking water of MRL+/+ mice for 40 weeks. Unlike TCE, TCAH did not induce AIH. Instead the TCAH-treated mice developed autoimmune alopecia complete with dose-dependent hair loss and skin inflammation [84]. Although TCAH induced a different disease outcome than TCE, TCAH stimulated T cell alterations identical to that of TCE, namely, an increase in CD44^hi^ IFN-*γ*-secreting CD4^+^ T cells. Thus, it appeared that its metabolite TCAH mediated the T cell effects of TCE, but the parent compound was required for those T cell effects to target the liver. 

In trying to understand the role of the parent compound in TCE-induced AIH, it is important to take into account that not all of the TCE-O-P450 formed by CYP2E1 is immediately metabolized to TCAA; some of it act as a reactive intermediate. The amino group of lysine on proteins reacts with intermediates formed during the hydrolysis of the TCE oxide forming N^6^-formyl lysine or N^6^-dichloroacetyllysine adducts. Antibodies to the dichloroacetyl lysine adducts [85] were used to detect these adducts as stable neoantigens in the liver of TCE-treated MRL+/+ mice [83]. One of the primary dichloroacetyl lysine-adducted protein was found to be CYP2E1, the main enzyme for TCE oxidative metabolism. It is possible that the formation of these neoantigens (liver proteins altered by TCE-O-P450) is required for TCE-induced AIH.

## 8. TCE and CD4^+^ T Cell Apoptosis

Experiments were conducted to determine how exposure to TCE or TCAH expanded the population of activated CD4^+^ T cells in the MRL+/+ mice. The investigation focused on Fas-mediated activation-induced cell death (aka restimulation-induced cell death), a process that is supposed to protect against the expansion of autoreactive CD4^+^ T cells [86–88]. The importance of this process in protection against autoimmunity is underlined by the finding that deficiencies in Fas-mediated apoptosis are found in a variety of idiopathic autoimmune diseases [89–96]. In terms of AIH, susceptibility to this disease has been linked to Fas polymorphisms, although the functional effects of these polymorphisms on activation-induced CD4^+^ T cell apoptosis in this disease has not been studied [33].

As demonstrated by Blossom et al. [97], 88% of the CD4^+^ T cells isolated from control MRL+/+ mice after a 4-week exposure to water alone were induced to undergo activation-induced apoptosis *in vitro*. In contrast, only 55% of CD4^+^ T cells from mice exposed to 0.5 mg/mL of TCE for 4 weeks underwent apoptosis. Exposure to TCAH *in vivo* for 4 or 40 weeks was similarly shown to suppress activation-induced apoptosis in CD4^+^ T cells [84]. The TCAH-induced decrease in apoptosis correlated with the decreased expression of FasL, but not Fas, on the surface of the CD4^+^ T cells. Subsequent experiments indicated that TCAH induced a mediated cleavage of FasL rather than suppressing FasL at the level of gene transcription [98]. Because sFasL is much less efficient than membrane-bound FasL at inducing Fas-mediated apoptosis in T cells, any mechanism that promotes FasL shedding can dramatically decrease FasL bioactivity [99, 100] thereby decreasing Fas-mediated apoptosis and promoting CD4^+^T cell-mediated autoimmunity.

## 9. Metalloproteinases and AIH

Metalloproteinases are a large group of proteases that include two families known as the matrix metalloproteinases (MMPs) and “a disintegrin and metalloproteinases” (ADAMs). MMPs are primarily secreted enzymes that have a variety of functions, including cleavage of cell-surface molecules and promoting the release of growth factors and cytokines [101, 102]. ADAMs are functionally similar to MMPs but, unlike most MMPs, are primarily cell-surface proteins. 

The role of metalloproteinases in AIH specifically is not known. However, these enzymes have been implicated in several types of autoimmune diseases as well as chronic liver diseases. For example, MMP-7 was shown to be one of the best discriminators between cirrhosis and precirrhotic stages in patients with chronic active hepatitis C [103]. Similarly, increased mRNA levels of MMP-9 or MMP-2 have been found in the livers of patients with chronic viral hepatitis as well as nonalcoholic steatohepatitis [104]. The functional importance of metalloproteinases in liver disease was underscored by the finding that a general metalloproteinase inhibitor blocked lethal hepatitis induced by TNF-*α* treatment in mice [105]. In terms of autoimmune disease several metalloproteinases including ADAM-17, MMP-9, MMP-3, MMP-2, or MMP-8 are reportedly increased in the serum or peripheral leukocytes of patients with systemic sclerosis, system lupus erythematosus, and Wegener's granulomatosis [106–109]. In mouse models of rheumatoid arthritis and experimental autoimmune uveoretinitis, the use of specific or general metalloproteinase inhibitors were shown to inhibit disease pathology [110, 111]. It still remains to be seen whether metalloproteinases play a direct role in initiating autoimmune diseases and certain types of liver pathology, or whether they are upregulated as a consequence of disease development and are a secondary consequence of tissue repair and/or inflammation.

## 10. Working Model of TCE-Induced AIH

**Figure 2:**
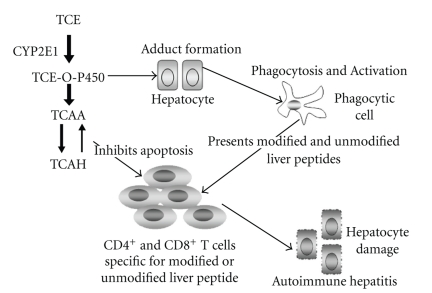
Model of TCE-induced AIH.

Taken together, the results obtained thus far have led to a model in which TCE-induced AIH in MRL+/+ mice requires two related mechanisms (Figure 2). In one mechanism TCE, via its metabolite TCAH, increases metalloproteinase activity. The TCAH-induced metalloproteinase activity cleaves FasL from the surface of activated CD4^+^ T cells, thus inhibiting their susceptibility to Fas-mediated activation-induced apoptosis. In a second mechanism TCE, through the generation of the TCE-O-P450 active metabolite, generates liver adducts. The adduct formation results in the activation of CD4^+^ T cells specific for both chemically modified and unmodified liver peptides. Because of their decreased susceptibility to apoptosis, the CD4^+^ T cells activated in response to either modified or unmodified liver proteins escape deletion but retain pathogenic effector function. 

Many aspects of this model need to be delineated. For example, the cellular source of the TCAH-induced metalloproteinase activity is not yet known. Also unclear is the molecular basis for the stimulatory effects of TCAH. Studies have demonstrated that TCAH in its aldehyde form can trigger signaling events with T cell surface molecules through the generation of a chemical reaction known as a Schiff base [112]. The nature of these signaling events and the identification of the cell surface molecules involved remain to be determined. Similarly, we still need to test that the signaling events associated with Schiff base formation are sufficient to trigger metalloproteinase activity. Although this model is described for TCE, it could also apply to other toxicants with reactive intermediates and with metabolites that exist as Schiff base-forming aldehydes.

## Figures and Tables

**Table 1:** Revised scoring system of the international autoimmune hepatitis group.

Parameter	Factor	Score
Gender	Female	+2
Alk Phos: AST (or ALT) ratio	>3	−2
	<1.5	+2
Gamma-globulin or IgG level above normal	<1.0 to > 2.0	0 to +3*
ANA, SMA, or anti-LKM1 titers	<1:40 to >1:80	0 to +3*
AMA	Positive	−4
Viral markers of active infection	Positive	−3
	Negative	+3
Hepatotoxic drugs	Yes	−4
	No	+1
Alcohol	<25 g/d	+2
	>60 g/d	−2
Concurrent immune disease	Thyroiditis, colitis, other	+2
Other autoantibodies	Anti-SLA, -actin, -LC1, -pANCA	+2
Histologic features	Interface hepatitis	+3
	Plasma cells	+1
	Rosettes	+1
	None of above	−5
	Biliary changes	−3
	Atypical features	−3
HLA	DR3 or DR4	+1
Treatment response	Remission alone	+2
	Remission with relapse	+3
Pretreatment aggregate score		
Definite diagnosis		>15
Probable diagnosis		10–15
Posttreatment aggregate score		
Definite diagnosis		>17
Probable diagnosis		12–17

*Depends upon titer. Alk phos: serum alkaline phosphatase level; AST: aspartate aminotransferase; ALT: alanine aminotransferase; IgG: immunoglobulin G; ANA: antinuclear antibody; SMA: smooth muscle antibody; LKM: liver/kidney microsomes; SLA: soluble liver antigen; LC1: liver cytosol type 1; pANCA: perinuclear anti-neutrophil cytoplasmic antibody; HLA: human leukocyte antigen.

## Abbreviations

AIH: Autoimmune hepatitis
TCE: Trichloroethylene
TCAH: Trichloroacetaldehyde hydrate
IFN-*γ:*: Interferon-gamma
ANA: Antinuclear antibodies
SMA: Smooth-muscle antibodies
ANCA: Antineutrophil cytoplasmic autoantibodies
SLA/LP: Soluble liver antigens or liver-pancreas
LKM-1: Liver-kidney microsomal-1
LC1: Liver cytosol 1
ASGPR: Asialoglycoprotein receptor
ADH: Alcohol dehydrogenase
TCR: T cell receptor
HLA: Human leukocyte antigen
CYP: Cytochrome P450
CCl_4_: Carbon tetrachloride
MMP: Matrix metalloproteinase
ADAM: A disintegrin and metalloproteinases.
